# Predicting prognosis using a pathological tumor cell proportion in stage I lung adenocarcinoma

**DOI:** 10.1111/1759-7714.14427

**Published:** 2022-04-13

**Authors:** Hyun Woo Jeon, Young‐Du Kim, Sung Bo Sim, Mi Hyoung Moon

**Affiliations:** ^1^ Department of Thoracic and Cardiovascular Surgery, Bucheon St. Mary's Hospital, College of Medicine The Catholic University of Korea Seoul Republic of Korea; ^2^ Department of Thoracic and Cardiovascular Surgery, Seoul St. Mary's Hospital, College of Medicine The Catholic University of Korea Seoul Republic of Korea

**Keywords:** adenocarcinoma, pathological tumor cell size, stage I, tumor volume

## Abstract

**Background:**

Tumor size is a valuable prognostic factor because it is considered a measure of tumor burden. However, it is not always correlated with the tumor burden. This study aimed to identify the prognostic role of pathological tumor proportional size using the proportion of tumor cells on the pathologic report after curative resection in pathologic stage I lung adenocarcinoma.

**Methods:**

We retrospectively reviewed the medical records of 630 patients with pathologic stage I lung adenocarcinoma after lung resection for curative aims. According to the pathologic data, the proportion of tumor cells was reviewed and pathological tumor proportional size was estimated by multiplying the maximal diameter of the tumor by the proportion of tumor cells. We investigated the prognostic role of pathological tumor proportional size.

**Results:**

The median tumor size was 2 cm (range: 0.3–4), and the median pathological tumor proportional size was 1.5 (range: 0.12–3.8). This value was recategorized according to the current tumor‐node‐metastasis (TNM) classification, and 184 patients showed down staging compared with the current stage. The survival curve for disease‐free survival using pathological tumor proportional size showed more distinction than the current stage classification. Multivariate analysis revealed that a down stage indicated a favorable prognostic factor.

**Conclusion:**

Pathological tumor cell proportional size may be associated with prognosis in stage I lung adenocarcinoma. If the pathological tumor proportional size shows a downward stage, it may indicate a smaller tumor burden and better prognosis

## Graphical abstract

The entire tumor size is a valuable prognostic factor and plays a key role in determining cancer stage and treatment. It is considered tumor burden and is correlated with cancer progression. However, tumor size usually has no prognostic impact in stage I lung adenocarcinoma from many studies. In this study, we investigated the prognostic role of tumor size using tumor cell proportion.

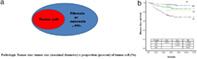

## INTRODUCTION

The incidence of lung cancer has been increasing and is the leading cause of death worldwide.^1^ Although low‐dose computed tomography (CT) contributes to the early detection of lung cancer^2^ and surgical resection is the most curative treatment,^3^ the therapeutic effect remains unsatisfactory compared with other malignancies. The 5‐year survival rate is ~70%–90% in stage I lung cancer.^4^ In stage I lung cancer, most patients underwent surgery for curative aims. Prognostic analysis is very important for predicting recurrence, and tumor‐node‐metastasis (TNM) classification is the best model to guide the appropriate treatment for patients with lung cancer. TNM classification has been updated continuously. In the T‐category, entire tumor size is the most important factor. It represents the maximal tumor diameter and is easily measured in the preoperative and postoperative settings. T stage is more categorized according to tumor size because it generally correlates with aggressiveness and prognosis.^5^ However, ground glass opacity (GGO) has been increasing and indicates favorable prognosis despite the size in the early stage of lung adenocarcinoma, and tumors are generally irregular.

In particular, invasive tumor size is more important than entire tumor size in GGO predominant adenocarcinoma. This means that the entire tumor size may not correlate with tumor burden and that real tumor cell volume (TV) may be expected to be smaller than tumor size in lung adenocarcinoma. Tumor volume may indicate the extent of the tumor burden more exactly than tumor size.^6^ We speculate that TV is a more reliable prognostic factor than tumor size in stage I lung adenocarcinoma. However, estimation of TV is very difficult in the postoperative setting. Furthermore, cancer is not always composed of only tumor cells. Tumor may include cancer cells, fibrotic lesion, and normal structures. We hypothesized that the tumor cell proportion is more associated with tumor burden. We believe that pathological tumor cell proportional size (PTS) affects prognosis as a replacement value of TV. This study aimed to investigate whether PTS is associated with prognosis after surgical resection for stage I lung adenocarcinoma.

## MATERIALS AND METHODS

We retrospectively reviewed the electronic medical records (EMRs) of patients who underwent surgical resection for lung cancer from January 2010 to April 2016. The Institutional Review Board of the Catholic Medical Center (XC21RADI0048) approved the study, and written informed consent from the patients was waived because the study was a retrospective analysis of the data. All the patients were classified with the 8th edition of the TNM classification. Patients with pathologic stage I lung adenocarcinoma were included. Patients with minimally invasive adenocarcinoma, multifocal GGO, neoadjuvant treatment, incomplete resection, and missing records were excluded. For the surgical procedure, patients with wedge resection were excluded because it was unclear whether the purpose of wedge resection was for a curative aim or biopsy. Finally, 630 patients with pathologic stage I lung adenocarcinoma were reviewed.

Preoperative studies included blood sampling, including carcinoembryonic antigen (CEA), pulmonary function test (PFT), chest CT, positron emission tomography‐CT (PET‐CT), brain magnetic resonance imaging (MRI), bone scanning, and bronchoscopy. Echocardiography was carried out for the patients older than 60 years.

Most of the patients underwent lobectomy or segmentectomy with mediastinal lymph node dissection or sampling according to the surgeon's decision based on a preoperative study.^7^ If the tumor was a pure GGO or a GGO‐dominant lesion located in the central portion, segmentectomy or lobectomy without mediastinal lymph node (LN) evaluation was performed according to the surgeon's decision based on the preoperative imaging study.

**FIGURE 1:**
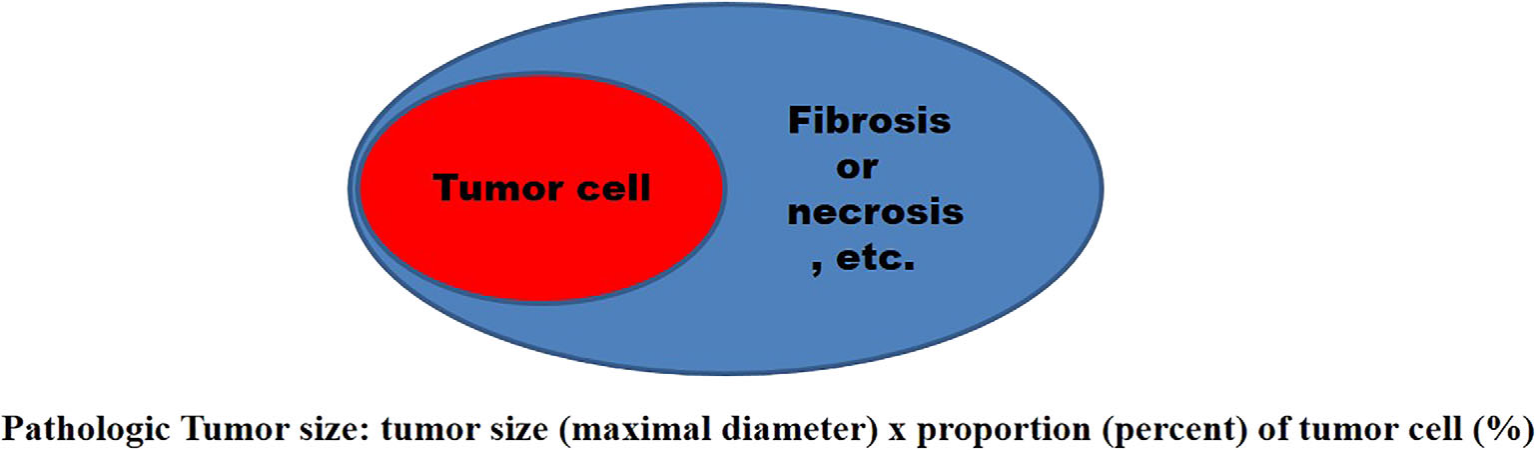
Pathological tumor cell proportional size calculation

We reviewed the pathologic data. The proportion of tumor cell was reviewed. The pathologic data demonstrated the maximal tumor diameter and tumor cell percentage (Figure 1). PTS was calculated by multiplying the maximal diameter of the tumor by the percentage of tumor cells (PTS = entire tumor size × percentage of tumor cells). T‐staging was reclassified using the calculated value of PTS.

We also reviewed the histologic subtype (acinar, papillary, micropapillary, lepidic, solid, and variants) proposed by the International Association for the Study of Lung Cancer, the American Thoracic Society, and the European Respiratory Society (IASLC/ATS/ERS) in 2011.^8^

Median follow‐up period was 72 months (range 1–125). Follow‐up (F/U) was conducted every 3 months for 1 year after the operation, every 4 months in the second year and every 6 months thereafter. Chest CT evaluation was conducted on every visit. If the recurrence was suspicious, further evaluation was carried out including PET CT or biopsy. All patients were followed until recurrence and death or loss of F/U. Recurrence was defined as local or extrathoracic metastasis based on clinical and pathologic evidence.

### Statistical analysis

All statistical analyses were carried out using SPSS version 18 (SPSS). Continuous variables were compared using the Mann–Whitney *U*‐test, and categorical variables were compared using the χ^2^ test and Fisher's exact test.

Survival curves were generated by the Kaplan–Meier method and log‐rank test between the current and reclassified staging systems. We analyzed the receiver operating characteristic (ROC) curves using the current and reclassified staging systems and compared the prognostic discrimination by the area under the curve (AUC) values between the two models.

Prognostic factors associated with recurrence were determined using the Cox proportional hazards model after checking the proportionality assumption. Variables with *p* values <0.05 in the univariate analysis were included in the multivariate analysis.

## RESULTS

**FIGURE 2:**
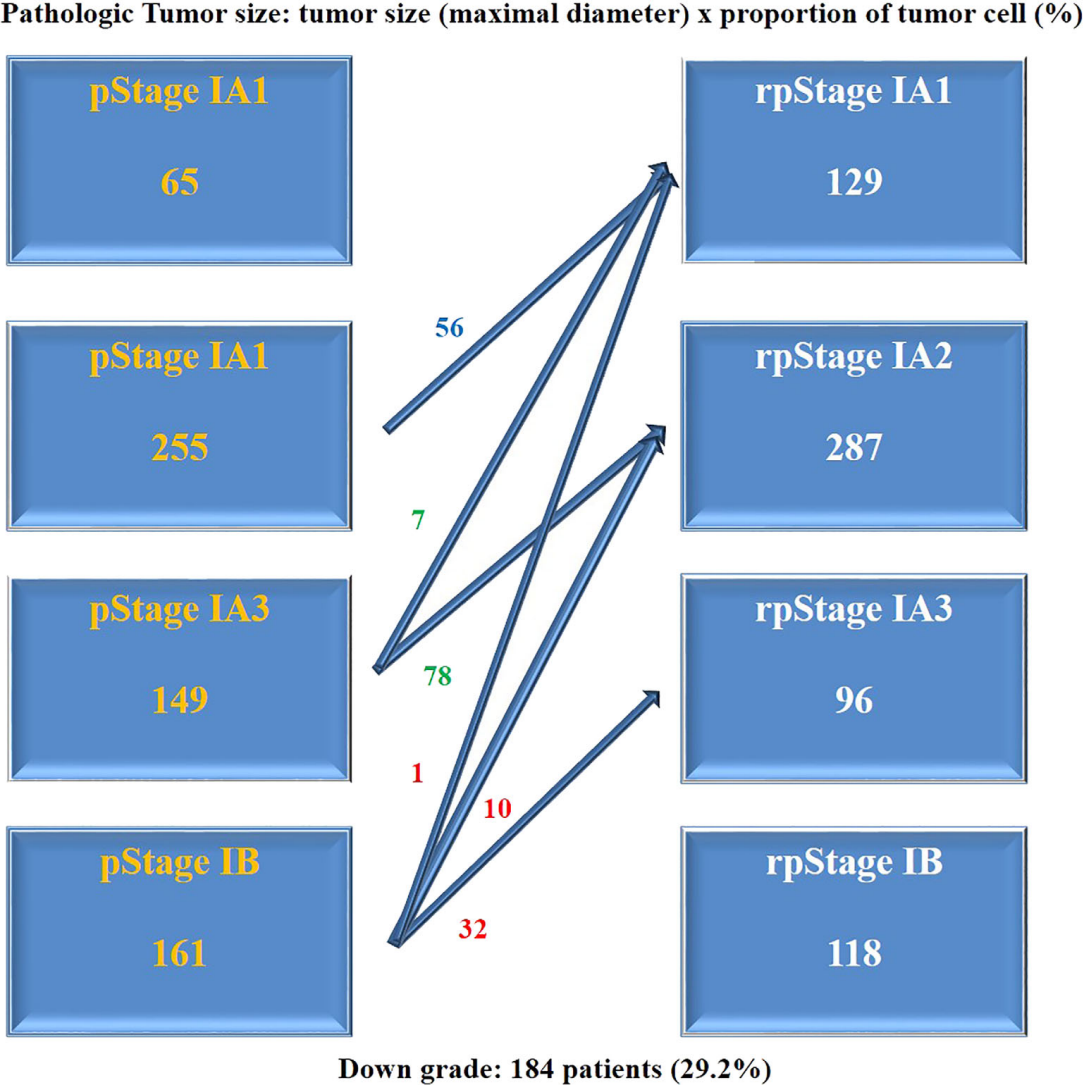
TNM re‐staging using a pathological tumor cell proportional size. Total 184 patients showed stage migration

The characteristics of the patients are shown in Table 1. There were 271 male patients (43%). A total of 359 patients had a history of smoking or current smoking at the time of the operation (57%). The median value of CEA and maximum standardized uptake value (SUVmax) were 1.5 (0.05–25.83) and 2.22 (0–18.5), respectively. Segmentectomy was conducted in 61 patients (9.7%). Video‐assisted thoracic surgery (VATS) was performed in 531 patients (84.3%). For the pathologic data, the median entire tumor size was 2 cm (range, 0.3–4), and the PTS was 1.5 cm (range, 0.12–3.8). Well differentiation was the most common (49.8%), and poor differentiation was only 8.1%. According to the pathological histologic subtype, the acinar predominant subtype was the most common (48.6%), and the lepidic predominant subtype was in 227 patients (36%). A total of 54 patients were the papillary predominant subtype (8.6%). The micropapilla (MP) (1.4%) and solid predominant subtype (4.6%) accounted for <5% of the patients. Variant type was found in five cases. Lymphovascular invasion (LVI) was identified in 172 patients (27.3%). Visceral pleural invasion (VPI) was identified in 103 patients (16.3%). There were 65 (10.3%), 255 (40.5%), 149 (23.7%), and 161 patients with pathologic stages IA1, IA2, IA3, and IB, respectively. Using a PTS, TNM staging was reconducted (Figure 2). If the patients had visceral pleural invasion, these patients were categorized as IB according to the current staging system regardless of the PTS. According to the TNM stage using a PTS, pathologic stage IA1 was 129 patients (20.5%). Stage IA2 included 287 patients (45.6%), and stage IA3 included 96 patients (15.2%). Stage IB was 118 patients (18.7%). A total of 184 patients showed down stage (29.2%).

**TABLE 1:** Baseline patient characteristics

Characteristic	Total (*n* = 630)
	Median (range) or no. (%)
Age, y	64 (25–86)
Male	271 (43.0)
Smoking	359 (57.0)
CEA	1.5 (0.05–25.83)
PET SUVmax	2.22 (0–18.5)
Procedure	
Bilobectomy	3 (0.5)
Lobectomy	566 (89.8)
Segmentectomy	61 (9.7)
VATS	531 (84.3)
Tumor size	2 (0.3–4)
Pathological tumor cell proportional size	1.5 (0.12–3.8)
Differentiation	
Well	314 (49.8)
Moderately	265 (42.1)
Poorly	51 (8.1)
Predominant histologic subtype	
Acinar	306 (48.6)
Lepidic	227 (36)
Papillary	54 (8.6)
Micropapillary	9 (1.4)
Solid	29 (4.6)
Margin	
Visceral pleural invasion	103 (16.3)
Lymphovascular invasion	172 (27.3)
pStage IA1	65 (10.3)
pStage IA2	255 (40.5)
pStage IA3	149 (23.7)
pStage IB	161 (25.6)

*Note*: Data are presented as the median (minimum‐maximum) or frequencies and percentages as appropriate.

Abbreviations: CEA, carcinoembryonic antigen; SUVmax, maximum standardized uptake value; VATS, video‐assisted thoracoscopic surgery.

The patients were divided into two groups according to stage migration (Table 2). Age, sex, and smoking history were not different between the two groups. Preoperative CEA was significantly higher in the group of stage migration (*p* < 0.001). There were significant differences in the entire tumor size and well differentiation between the two groups. In the stage migration group, the entire tumor size was larger, and well differentiation was less common. For the predominant histologic subtype, acinar and MP predominant subtypes were more common in the stage migration group (*p* = 0.018 and 0.021, respectively). LVI was also more prominent in the stage migration group (*p* < 0.001). However, recurrence was less common in the stage migration group (*p* = 0.029).

**TABLE 2:** The patients were divided according to the stage migration

Variables	Stage migration (−)	Stage migration (+)	*p* value
	*n* = 446	*n* = 184	
Age, y	63 (25–86)	64.7 (35–85)	0.051
Gender (male)	188 (42.2)	83 (45.1)	0.536
Smoking	126 (28.3)	45 (24.5)	0.375
CEA	1.41(0.05–19.15)	1.96 (0.5–25.83)	<0.001
PET SUVmax	2.15 (0–18.5)	2.45 (0–17.6)	0.058
Size	1.82 (0.3–4)	2.24 (1.1–4)	<0.001
PTS	1.46 (0.12–3.8)	1.54 (0.14–2.97)	0.247
Well differentiation	235 (52.7)	79 (42.9)	0.029
Predominant subtype			
&Acinar	203 (45.5)	103 (56)	0.018
&Lepidic	170 (38.1)	57 (31)	0.101
&Papillary	45 (10.1)	9 (4.9)	0.041
&MPP	3 (0.7)	6 (3.3)	0.021
&Solid	24 (5.4)	5 (2.7)	0.208
&LVI	99 (22.2)	73 (40)	<0.001
Recurrence	69 (14.8)	16 (8.7)	0.029

*Note*: Data are presented as the median (minimum‐maximum) or frequencies and percentages as appropriate.

Abbreviations: CEA, carcinoembryonic antigen; LVI, lymphovascular invasion; PTS, pathological tumor cell proportional size; MPP, micropapillary predominant; SUVmax, maximum standardized uptake value.

**FIGURE 3:**
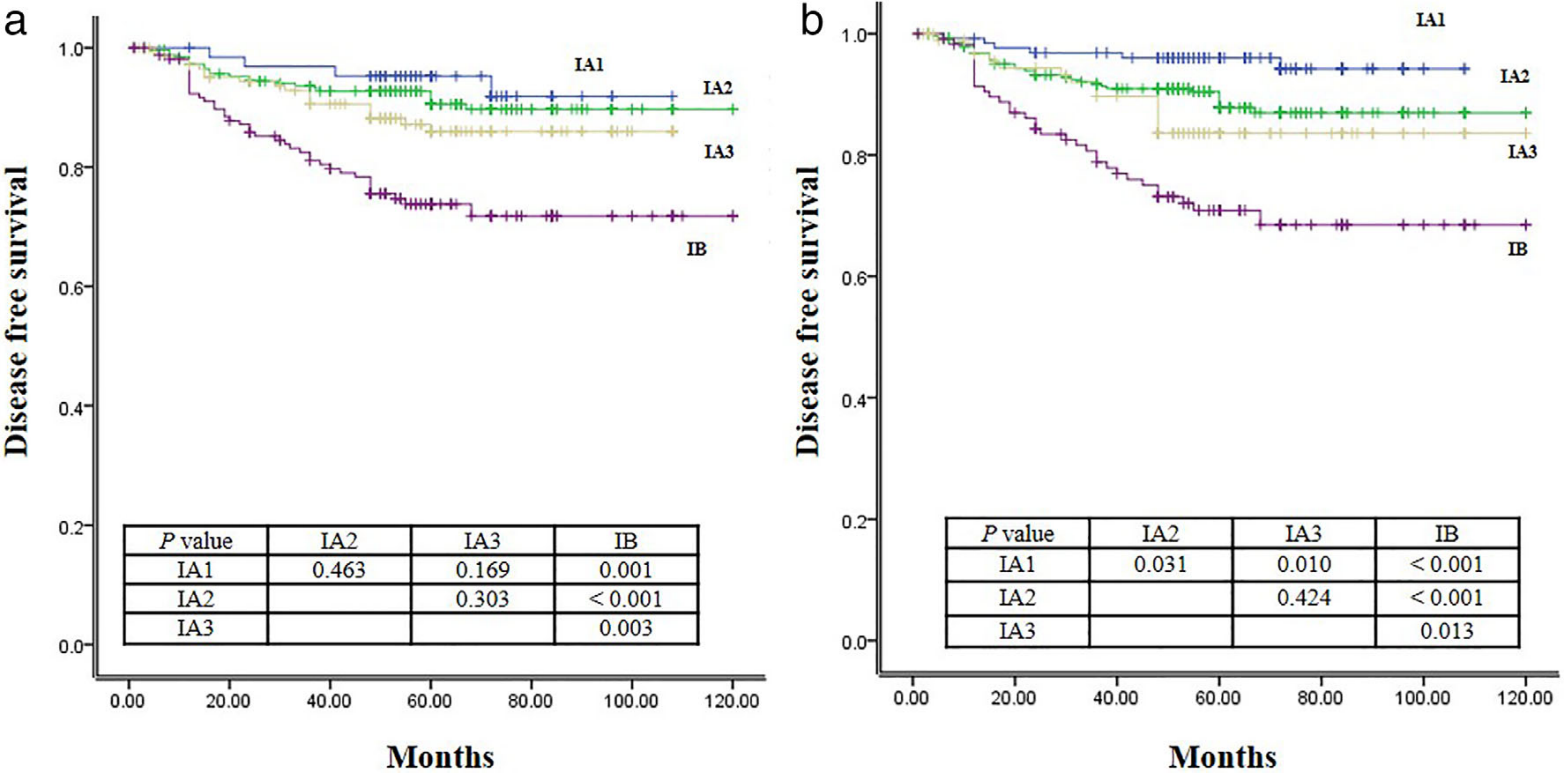
Survival curve for disease free survival according to the current TNM stage (a) and TNM re‐stage by a pathological tumor cell proportional size (b)

A survival curve for recurrence was obtained using the entire tumor size and PTS (Figure 3). There was a significant difference between stage IA and IB using the entire tumor size. However, there was no difference between stages IA1, IA2, and IA3 (Figure 3(a)). However, the survival curve according to PTS was more distinct than that according to tumor size (Figure 3(b)). Although there was no significant difference between IA2 and IA3, other stages were significantly different from each other.

**FIGURE 4:**
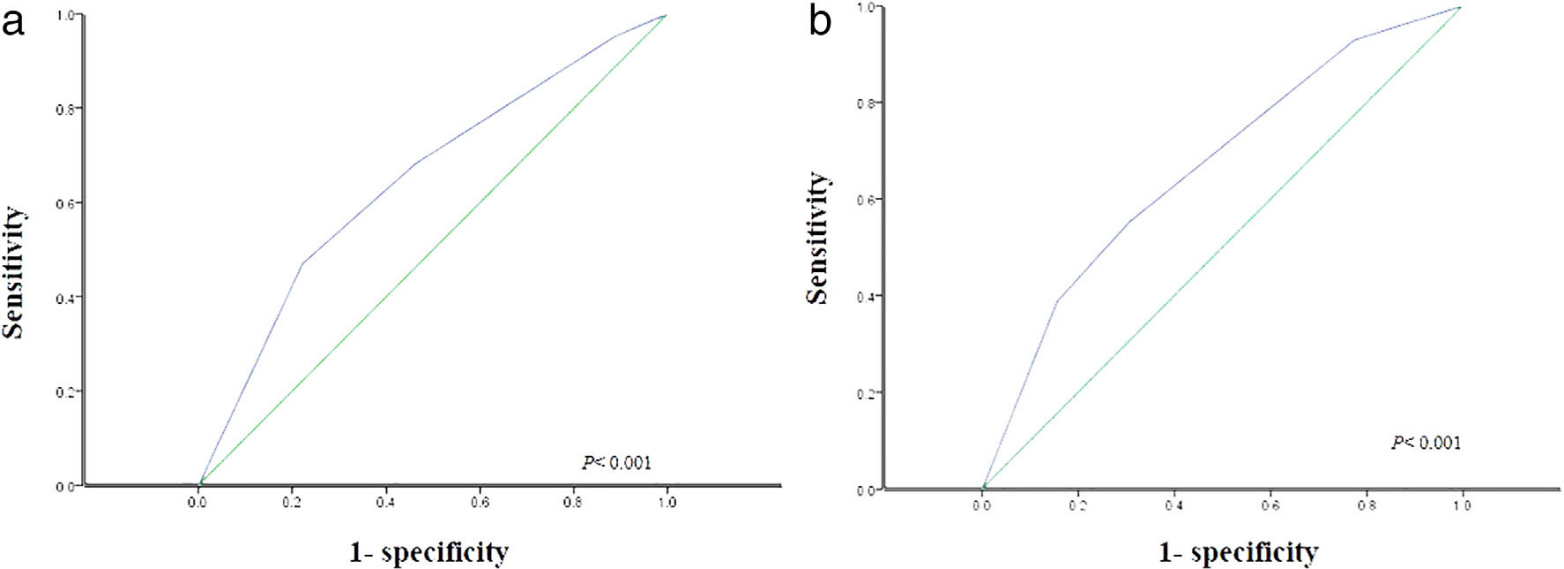
The area under the receiver operating characteristic curve for recurrence according to the current TNM stage (a) and TNM re‐stage by a pathological tumor cell proportional size (b)

According to the ROC curve for recurrence (Figure 4), the AUC of entire tumor size was 0.648 (95% CI, 0.584–0.712; *p* < 0.001). The AUS of PTS was 0.666 (95% CI, 0.604–0.727; *p* < 0.001). The value of AUC was improved using a PTS.

In univariate analysis for disease‐free survival (DFS), male sex (*p* = 0.016), smoking history (*p* < 0.001), CEA (*p* = 0.003), SUVmax (*p* < 0.001), entire tumor size (*p* < 0.001), PTS (*p* = 0.015), poor differentiation (*p* < 0.001), MP predominant subtype (*p* < 0.001), solid predominant subtype (*p* < 0.001), VPI (*p* < 0.001), close margin (*p* = 0.008), LVI (*p* < 0.001), and negative downstage migration (*p* = 0.043) were significant. SUVmax (*p* = 0.050), MP predominant subtype (*p* < 0.001), solid predominant subtype (*p* = 0.027), LVI (*p* < 0.001), and being negative for down stage migration (*p* = 0.003) were significant prognostic factors for DFS by multivariate analysis (Table 3).

**TABLE 3:** Univariate and multivariate analysis for disease free survival

Variables	Univariate			Multivariate		
	HR	95% CI	*p* value	HR	95% CI	*p* value
Male	1.689	1.102–2.588	0.016	1.433	0.796–2.582	0.231
Smoking	2.501	1.630–3.837	<0.001	1.678	0.940–2.995	0.080
CEA	1.088	1.029–1.151	0.003	1.066	0.984–1.155	0.119
SUVmax	1.186	1.135–1.240	<0.001	1.061	1.000–1.127	0.050
Tumor size	1.690	1.315–2.172	<0.001	1.354	0.832–2.203	0.222
PTS	1.430	1.071–1.910	0.015	0.710	0.425–1.184	0.189
Poorly differentiation	3.060	1.749–5.354	<0.001	0.842	0.422–1.681	0.626
MPP predominant	7.076	3.079–16.258	<0.001	6.740	2.529–17.959	<0.001
Solid predominant	4.855	2.573–9.159	<0.001	2.440	1.106–5.380	0.027
VPI	3.360	2.159–5.230	<0.001	1.422	0.799–2.530	0.231
Margin	0.827	0.719–0.952	0.008	0.916	0.785–1.070	0.268
LVI	4.288	2.786–6.599	<0.001	3.594	2.191–5.896	<0.001
Down stage (−)	1.752	1.017–3.018	0.043	2.927	1.427–6.003	0.003

Abbreviations: CEA, carcinoembryonic antigen; LVI, lymphovascular invasion; PTS, pathological tumor cell proportional size; MPP, micropapillary; SUVmax, maximum standardized uptake value; VPI, visceral pleural invasion.

Age (*p* = 0.006), male sex (*p* = 0.004), smoking history (*p* < 0.001), CEA (*p* = 0.004), SUVmax (*p* < 0.001), poor differentiation (*p* = 0.009), solid predominant subtype (*p* < 0.001), LVI (*p* = 0.001), and negative downstage migration (*p* = 0.040) were significant for overall survival (OS) in univariate analysis. SUVmax (*p* = 0.024), LVI (*p* = 0.011) and negative downstage migration (*p* = 0.010) were prognostic factors for OS by multivariate analysis (Table 4).

**TABLE 4:** Univariate and multivariate analysis for overall survival

Variables	Univariate			Multivariate		
	HR	95% CI	*p* value	HR	95% CI	*p* value
Age	1.051	1.014–1.089	0.006	1.032	0.997–1.069	0.073
Male	2.544	1.358–4.765	0.004	1.630	0.737–3.606	0.228
Smoking	3.099	1.702–5.642	<0.001	1.700	0.789–3.665	0.176
CEA	1.113	1.035–1.196	0.004	1.069	0.959–1.191	0.228
SUVmax	1.190	1.116–1.270	<0.001	1.101	1.013–1.198	0.024
Poorly differentiation	2.937	1.303–6.619	0.009	1.022	0.392–2.664	0.965
Solid	5.456	2.292–12.986	<0.001	2.005	0.697–5.768	0.197
VPI	1.981	0.997–3.935	0.051	0.900	0.425–1.904	0.782
LVI	2.757	1.501–5.066	0.001	2.449	1.228–4.880	0.011
Down stage (−)	2.476	1.045–5.867	0.040	3.460	1.341–8.929	0.010

Abbreviations: CEA, carcinoembryonic antigen; SUVmax, maximum standardized uptake value; VPI, visceral pleural invasion, LVI: lymphovascular invasion.

## DISCUSSION

Well‐established prognostic factor analysis is very important to determine effective treatment in the field of cancer medicine. The TNM staging system has been widely accepted for the treatment of lung cancer. The TNM staging classification for lung cancer has been updated by the International Association for the Study of Lung Cancer (IASLC) group. Entire tumor size is a valuable prognostic factor and plays a key role in determining cancer stage.^5^ Generally, it is considered tumor burden and is correlated with cancer progression. In particular, it is very easy and simple to measure in the preoperative and postoperative settings. The T category using the TNM staging system is a valuable parameter regarding prognosis in the early stage of lung adenocarcinoma.^9^ According to the current TNM staging system, the T1 category was divided into three subcategories (T1a, T1b, and T1c) according to the entire tumor size regardless of the cancer features, including GGO or partial solid nodules.^5^ The subcategory reflects prognosis for 5‐year OS (90% for 1a, 85% for 1b, and 73% for 1c).

However, it did not always reflect the tumor burden.^10^ In a previous study, although numerous studies have not been conducted, tumor volume may be a more reliable prognostic factor than tumor size because it reflects tumor burden more accurately, and advances in imaging techniques make it easy to measure the tumor volume.^11^ Takenaka et al.^12^ investigated whether tumor volume may be associated with prognosis in clinical stage IA lung cancer. They found that the entire tumor volume and solid portion volume were significant prognostic factors for DFS. Hyun et al.^13^ investigated prognostic factors in pathological early‐stage lung cancer. They used volume‐based parameters of ^18^F‐fluorodeoxyglucose PET‐CT and demonstrated that metabolic tumor volume is a more reliable prognostic factor than SUVmax in DFS and OS. Su et al.^14^ also investigated the prognostic impact of tumor volume on early‐stage lung cancer, and tumor volume was an independent prognostic factor for DFS and OS. However, there were some problems in determining the prognostic role of tumor volume and entire tumor size.

First, tumor volume measurements could only be conducted in the preoperative setting, so it represents clinical stage, not pathologic stage. When treatment plans have to be conducted, the pathological stage has to be widely accepted. However, pathological tumor volume measurements are very difficult. Furthermore, the resected lung was deflated after curative resection. This makes it difficult to measure tumor volume and real tumor size. Pathological tumor size may be smaller than clinical tumor size,^15^ and Travis et al.^16^ demonstrated that lepidic predominant tumor size was smaller than actual tumor size.

Second, tumor burden may not be associated with real tumor volume or entire tumor size because tumors include air space, fibrosis, or necrosis, and GGO is increased in lung adenocarcinoma.^17^ GGO is the lesion that has increased attenuation with bronchial and vascular structural preservation. Tumor cells were present along the alveolar walls. Hattori et al.^18^ evaluated patients with clinical N0M0 lung cancer. They found that tumor size was a significant prognostic factor in solid lung cancer. However, tumor size did not affect the prognosis of GGOs or part‐solid nodules.^18^ They suggested that GGOs and part‐solid nodules should be described as clinical Tis or T1a. Another previous study demonstrated that the size of the solid component is more important to determine prognosis than the entire tumor size.

We thought that we might need other parameters to add current T‐descriptors regarding prognosis in the early stage of lung adenocarcinoma.

We used tumor proportion in the specimen. Although it was not the actual value, we estimated the virtual value, calculated by multiplying the maximal diameter of the tumor by the proportion of tumor cells. We found a trend for prognosis according to the pathological tumor proportional size. According to the current TNM staging system in our study, there was a significant difference between IA and IB (*p* < 0.001). However, significance was not identified among the stage IA subcategory in our study. According to the PTS, the difference in the survival curve regarding DFS became more distinct, reflecting a significant difference in DFS except for DFS between IA2 and IA3.

The IASLC demonstrated that invasive size is more important in lung adenocarcinoma and recommended the measurement of the invasive size if the lesion is multi‐focal.^19^ The formula is that multiplying of percentage of invasive component area by the overall tumor size. This concept is very similar with us.

We evaluated the prognosis with stage migration compared to the current TNM staging system. A total of 184 patients showed stage migration, and most of them migrated to the last stage (IA2 → IA1, IA3 → IA2) because tumor cell size was decreased using a PTS. Univariate analysis for DFS revealed that numerous prognostic factors were identified, which are well‐known prognostic factors in lung adenocarcinoma. Multivariate analysis showed that SUVmax (*p* = 0.050), MP predominant subtype (*p* < 0.001), solid predominant subtype (*p* = 0.027), LVI (*p* < 0.001), and being negative for stage migration (*p* = 0.003) were significant prognostic factors.

Survival analysis for OS revealed that SUVmax (*p* = 0.024), LVI (*p* = 0.011) and being negative for stage migration (*p* = 0.010) were significant statistically.

The most interesting thing was that well‐known prognostic factors were more common in the stage migration group. CEA was higher, acinar, and MP predominant subtypes were more common in the stage migration group, and LVI was significantly higher in the stage migration group. However, recurrence was significantly lower. We could conclude that the stage migration group might have a tumor burden much smaller than the entire tumor size, so they showed a better prognosis than the non‐stage migration group.

There were several limitations in this study. First, this study was nonrandomized and retrospective in design with a relatively short F/U period for analyzing DFS and OS. In stage I lung adenocarcinoma, numerous adjuvant treatments increase survival. Second, the value of PTS was not the actual value. It was difficult to obtain the actual value because the specimen was deflated and the tumor margin was irregular. Knowing the limitations of the measurement method, we wanted to see the overall trend using tumor burden not entire tumor size and we found that decreased tumor burden compared with entire tumor size showed better prognosis. Third, mediastinal lymph node evaluation was omitted in some patients. Mediastinal lymph node evaluation could be omitted in selective cases, including pure GGO‐ or GGO‐dominant lesions.^20^ However, it could affect the recurrence. Finally, this study was not conducted from multiple centers; therefore, selection bias may be inevitable.

## CONCLUSIONS

PTS is associated with DFS and OS for stage I lung adenocarcinoma after curative resection. Because tumor burden is an important prognostic factor, PTS combined with entire tumor size may provide a prognostic role for tumor burden in lung adenocarcinoma.

## CONFLICTS OF INTEREST

All authors declare that they have no conflicts of interest associated with this study.

## Data Availability

The data that support the findings of this study are available on request from the corresponding author.
